# Cluster randomised controlled trial of a peer-led lifestyle intervention program: study protocol for the Kerala diabetes prevention program

**DOI:** 10.1186/1471-2458-13-1035

**Published:** 2013-11-04

**Authors:** Thirunavukkarasu Sathish, Emily D Williams, Naanki Pasricha, Pilvikki Absetz, Paula Lorgelly, Rory Wolfe, Elezebeth Mathews, Zahra Aziz, Kavumpurathu Raman Thankappan, Paul Zimmet, Edwin Fisher, Robyn Tapp, Bruce Hollingsworth, Ajay Mahal, Jonathan Shaw, Damien Jolley, Meena Daivadanam, Brian Oldenburg

**Affiliations:** 1 School of Public Health and Preventive Medicine, Monash University, Melbourne, Australia; 2 National Heart and Lung Institute, Imperial College London, London, UK; 3 University of Tampere, Tampere, Finland; 4 Centre for Health Economics, Monash University, Melbourne, Australia; 5 Sree Chitra Tirunal Institute for Medical Sciences and Technology, Thiruvananthapuram, Kerala, India; 6 Baker IDI Heart and Diabetes Institute, Melbourne, Australia; 7 Department of Health Behavior, Gillings School of Global Public Health, Peers for Progress, American Academy of Family Physicians Foundation, University of North Carolina, Chapel Hill, USA; 8 Optometry and Vision Sciences, University of Melbourne, Melbourne, Australia; 9 Division of Health Research, Lancaster University, Lancaster, UK; 10 Department of Public Health Sciences, Karolinska Institutet, Stockholm, Sweden

**Keywords:** Diabetes, Incidence, India, Kerala, Peer support, Randomised controlled trial, Prevention, Resource-constrained settings, Rural, Intervention

## Abstract

**Background:**

India currently has more than 60 million people with Type 2 Diabetes Mellitus (T2DM) and this is predicted to increase by nearly two-thirds by 2030. While management of those with T2DM is important, preventing or delaying the onset of the disease, especially in those individuals at ‘high risk’ of developing T2DM, is urgently needed, particularly in resource-constrained settings. This paper describes the protocol for a cluster randomised controlled trial of a peer-led lifestyle intervention program to prevent diabetes in Kerala, India.

**Methods/design:**

A total of 60 polling booths are randomised to the intervention arm or control arm in rural Kerala, India. Data collection is conducted in two steps. Step 1 (Home screening): Participants aged 30–60 years are administered a screening questionnaire. Those having no history of T2DM and other chronic illnesses with an Indian Diabetes Risk Score value of ≥60 are invited to attend a mobile clinic (Step 2). At the mobile clinic, participants complete questionnaires, undergo physical measurements, and provide blood samples for biochemical analysis. Participants identified with T2DM at Step 2 are excluded from further study participation. Participants in the control arm are provided with a health education booklet containing information on symptoms, complications, and risk factors of T2DM with the recommended levels for primary prevention. Participants in the intervention arm receive: (1) eleven peer-led small group sessions to motivate, guide and support in planning, initiation and maintenance of lifestyle changes; (2) two diabetes prevention education sessions led by experts to raise awareness on T2DM risk factors, prevention and management; (3) a participant handbook containing information primarily on peer support and its role in assisting with lifestyle modification; (4) a participant workbook to guide self-monitoring of lifestyle behaviours, goal setting and goal review; (5) the health education booklet that is given to the control arm. Follow-up assessments are conducted at 12 and 24 months. The primary outcome is incidence of T2DM. Secondary outcomes include behavioural, psychosocial, clinical, and biochemical measures. An economic evaluation is planned.

**Discussion:**

Results from this trial will contribute to improved policy and practice regarding lifestyle intervention programs to prevent diabetes in India and other resource-constrained settings.

**Trial registration:**

Australia and New Zealand Clinical Trials Registry: ACTRN12611000262909.

## Background

Globally, India has the second largest number of individuals with Type 2 Diabetes Mellitus (T2DM) (63 million), and this is expected to rise to 101.2 million by 2030 [1]. While management of those already diagnosed with T2DM is important, preventing or delaying the onset of the disease, especially in those individuals at ‘high risk’ of developing T2DM, is needed to control the growth of the disease [2], particularly in resource-constrained settings [3]. Thus, the prevention of T2DM, through a combination of individual-, community- and population-based approaches needs urgent attention.

The efficacy of lifestyle interventions at preventing or delaying the onset of T2DM is well-established [4-11]. Aziz et al., reported around 30 implementation trials that have demonstrated a reduction in T2DM incidence of between 40-60% compared with the control arm. However, these trials have mainly been conducted in high-income countries [Aziz et al. 2013, unpublished data]. Indeed, the non-pharmacological and behavioural intervention methods used in these trials have been shown to be more cost-effective than drug treatment [12,13], particularly when delivered via groups [14].

In India, diabetes prevention trials conducted to date have primarily targeted those with impaired glucose tolerance (IGT) or impaired fasting glucose (IFG) [10,15]. However, in resource-constrained settings, it is important to use a risk tool that is valid, reliable, low cost, quick and easy to administer to identify the individuals at ‘high risk’ of developing T2DM rather than with the use of laboratory testing. The Indian Diabetes Prevention Programme (IDPP-1) was a three-year randomised controlled trial among an urban population with persistent IGT that implemented a lifestyle intervention (individualised advice by a health provider on healthy diet and regular physical activity, with monthly telephone calls to maintain motivation) [10]. The sample selected was predominantly middle class and from a restricted occupational setting. Although the study was effective and showed a reduction in T2DM incidence, the approach tested would be difficult to ‘scale up’ to community or national level, i.e. it is not feasible to provide one-on-one advice to the 21 million people with IGT in India [1]. Furthermore, more than two-thirds of India’s population lives in rural areas and effective approaches to diabetes prevention in this setting require strategies that are less dependent on health care providers and health care services. A lifestyle intervention program by Balagopal et al., conducted under more ‘real world’ conditions in a rural area, achieved an 11% reduction in fasting plasma glucose (FPG) levels in people with IFG [15]. However, the study did not include a control group and comprised small numbers with IFG.

To stem the growing epidemic of T2DM in India and other resource-constrained settings, evidence is required regarding cost-effective community-based approaches for screening and prevention that have high future scalability. In our previous research, we have developed and tested models for community interventions in Finland and Australia. We have been able to show that group-based approaches with underlying behaviour change theory and strong emphasis on peer support will lead to significant improvement in health behaviours and metabolic risk factors [16-19]. More broadly, substantial research from around the world shows the effectiveness of peer support in prevention and disease management [20-24] as well as engaging audiences whom health promotion programs often have difficulty reaching [25,26]. Adapted from these programs, the Kerala Diabetes Prevention Program (K-DPP) is the first implementation trial to evaluate a peer-led, group-based lifestyle intervention program among individuals at ‘high risk’ of developing T2DM in rural India.

## Methods/design

### Study aims

#### Primary

To evaluate the effectiveness of a peer-led, group-based lifestyle intervention on reducing T2DM incidence and improving the behavioural, psychosocial, clinical, and biochemical measures at 24 months, compared with the control arm.

#### Secondary

(i) To estimate the cost and cost-effectiveness of the intervention in reducing the T2DM incidence and improving quality of life.

(ii) To determine the reach, dose delivered, dose received and fidelity of the intervention.

(iii) To identify the individual-, household-, and neighbourhood-level factors likely to influence the scalability of K-DPP in India and other resource-constrained settings in the future.

### Study design and setting

**Figure 1:**
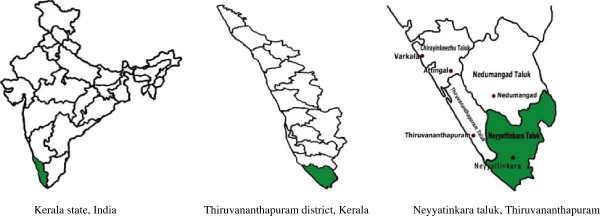
Study area of the Kerala diabetes prevention program.

**Figure 2:**
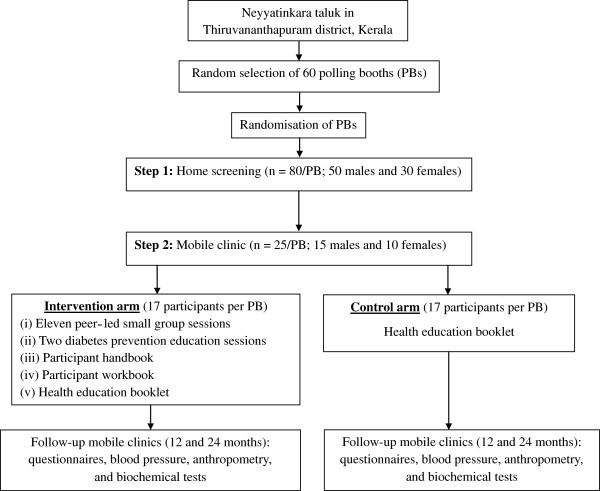
CONSORT diagram of the Kerala diabetes prevention program.

The study is a cluster randomised controlled trial, implemented and reported in accordance with the Consolidated Standards of Reporting Trials (CONSORT) statement [27] and its extension to cluster randomised trials [28]. The study is conducted in 60 polling booths (PBs) in Neyyatinkara *taluk* (taluk is the administrative unit below the district level in rural areas), Thiruvananthapuram district, in the state of Kerala. Figure 1 shows the study area of K-DPP. The epidemiological transition in Kerala is more advanced than elsewhere in India [29]; indeed it has the largest proportion of those with several major risk factors for non-communicable diseases (NCDs) [30,31]. The state has a population of 33.4 million (52.3% in rural areas) in a surface area of 38,863 square kilometers with a literacy rate of 93.9%, life expectancy of 74.6 years, and sex ratio of 1084 females per 1000 males [32]. Given the stage of epidemiological transition, Kerala is likely to be a ‘harbinger’ of what will happen in the future to the rest of India in terms of NCDs [33,34]. Therefore, Kerala provides an appropriate place to implement and evaluate a community-based diabetes prevention program in India. Figure 2 is a CONSORT diagram of the study design.

### Ethics approval

The study was approved by the Institutional Ethics Committee of the Sree Chitra Tirunal Institute for Medical Sciences and Technology, Thiruvananthapuram (SCT/IEC-333/May 2011) and the Human Research Ethics Committee of Monash University, Australia (CF11/0457-2011000194). The study was also approved by the Health Ministry Screening Committee of the government of India. Written informed consent is also obtained from all the study participants.

### Sample size calculations

The IDPP-1 study offers the best estimate for the incidence of T2DM in the control arm, 18.3% per year [10]. Our precision calculations are based on the Poisson distribution, allowing for an average of 15 participants per polling booth enrolled at baseline and followed-up after two years. An intra-class correlation coefficient for FPG measurements of 0.02 has been assumed [35]. For a significance level of 5% and Type II error of 20% (i.e. power = 80%), allowing a loss to follow-up of 10%, the numbers of participants and PBs per arm required are 510 and 30 respectively for a feasible and clinically significant relative risk of 0.70, i.e. reduction by 30% in the proportion of participants with incident T2DM after two years. Thus, our target recruitment is 60 PBs and 17 participants per PB.

### Sample selection procedures

#### Clusters

Neyyatinkara taluk has four legislative assembly constituencies (LACs) with 603 PBs. Unfortunately there was no map available that showed the contiguous PBs across the LAC borders, therefore PBs that lie along the borders of LACs (244) were removed and 60 PBs (15 from each LAC) were randomly selected from the remaining 359 PBs. The 15 PBs in each LAC were then randomised to the intervention arm or control arm. This was done by a biostatistician who was blind to all other characteristics of the sampled units and by using a constant block size and stratified by the size of PB (approximately 900–1500 people aged ≥18 years/PB), using Stata statistical software, Release 12. Contiguous PBs were replaced by the next PB to preclude the risk of contamination across boundaries of the PB.

#### Participants

The pilot study revealed that it is essential to approach more males than females because males tended to be unavailable during home screening (Step 1 of data collection) as they work away from home and their participation rate was low in mobile clinics (Step 2 of data collection). To obtain 17 participants per PB (approximately equal number of males and females), 80 individuals (50 males and 30 females) aged 30–60 years selected randomly from the electoral roll are approached during home screening. The electoral roll is in the public domain with details of name, age, sex, and address and is updated every five years. Of those screened and who meet the eligibility criteria, 25 individuals (15 males and 10 females) are invited to attend a mobile clinic. This recruitment strategy is based on three assumptions: 1) 20% (n = 16) of the 80 individuals will not meet the eligibility criteria at Step 1; 2) 57% (n = 37) of those eligible at Step 1 will not be classified as ‘high risk’ of having T2DM according to the Indian Diabetes Risk Score (IDRS) with a cut-off value of ≥60 [36]; and 3) Approximately 8 to 10 participants will be classified as having T2DM based on the sensitivity of IDRS (72.5%) [36] and the awareness of diabetes (63.7%) [33] and therefore excluded at Step 2.

### Study eligibility criteria

Eligible participants comprise randomly selected males and females on the electoral roll from the selected PBs, aged 30 to 60 years, and able to speak, read and write Malayalam (the local language). Participants are excluded if they have prior diagnosis of T2DM, myocardial infarction, heart failure, stroke, cancer, epilepsy, arthritis or dementia, or currently use medications known to affect glucose tolerance (glucocorticoids, anti-psychotic drugs and anti-retroviral drugs). Pregnant women are also excluded. Those at baseline diagnosed with T2DM, based on a 2 hour oral glucose tolerance test (OGTT) are excluded, and referred to a government healthcare facility for further management. Diabetes is diagnosed on the basis of fasting plasma glucose ≥ 126 mg% (7.0 mmol/L) and/or post glucose load ≥ 200 mg% (11.1 mmol/L) as per the World Health Organisation (WHO) criteria [37].

### Data collection

Data collection is conducted in four waves (each wave consists of one LAC with 15 PBs). There are two steps in data collection namely, home screening and mobile clinic. The data collectors are given training prior to the commencement of data collection and refresher training after each wave using a training manual developed in accordance with the WHO STEPS (Stepwise approach to surveillance) training guide [38]. Table 1 shows the measurement domains, tools and time points (baseline, 12 and 24 months) at which data are collected.

**Table 1:** Measurement domains, tools, and data collection time points (baseline, 12 months and 24 months)

**Variable**	**Component**	**Measurement tools/questions**	**Baseline**	**12 months**	**24 months**
Socio-demographic measures		Age, sex, marital status, education, religion, occupation, household size, and monthly household expenditure	✓	✓	✓
Behavioural measures	Tobacco use	WHO STEPS questionnaire [38]	✓	✓	✓
	Alcohol use	WHO STEPS questionnaire [38]	✓	✓	✓
	Physical activity	Global Physical Activity Questionnaire [39]	✓	✓	✓
	Sedentary behaviour	Time spent in front of a screen [40]	✓	✓	✓
	Diet	Food Frequency Questionnaire adapted from PROLIFE study [41]	✓	✓	✓
Diabetes knowledge	Barriers to healthy eating	Scale designed for trial	✓	✓	✓
	Barriers to physical activity	Adapted from the scale designed by Booth et al. [42]	✓	✓	✓
	Self-efficacy (diet and physical activity)	Adapted from the Nutrition and physical Activity self-efficacy scales designed by Schwarzer and Renner [43]	✓	✓	✓
Psychosocial measures	Depression	Patient Health Questionnaire-9 amended in line with CURES-65 study [44]	✓	✓	✓
	Stress	Chronic stress scale used in MESA study [45,46]	✓	✓	✓
	Anxiety	General anxiety disorder scale [47]	✓	✓	✓
	Health-related quality of life	Short Form-36 [48]	✓	✓	✓
	Social support	ENRICHD social support scale [49]	✓	✓	✓
	Life satisfaction	How satisfied are you with your life as a whole?	✓	✓	✓
Medical history		Use of any medications, family history of diabetes, heart disease or stroke, history of hypertension, and history of high cholesterol	✓	✓	✓
Clinical measures	Anthropometry	Waist circumference (Seca measuring tape) [38] Hip circumference [38]	✓	✓	✓
		Height (Seca stadiometer) [38]	✓	✓	✓
		Weight (TANITA body composition analyser) [38]	✓	✓	✓
		Bioimpedance (TANITA body composition analyser) [38]	✓	✓	✓
	Blood pressure	Omron automatic blood pressure monitor [38]	✓	✓	✓
Biochemical measures	Pathology	Glycaemic control (fasting plasma glucose and 2 hour post load glucose, HbA1c), lipid profile (total cholesterol, HDL, LDL, triglycerides), and fibrinogen	✓	✓	✓
Cost effectiveness analysis	Healthcare utilisation	Direct and indirect costs associated with outpatient and inpatient healthcare services, sources of financing, and time away from work due to ill health	✓	✓	✓
Program evaluation	Knowledge assessment	Pre and post test	✓		
	Training evaluation	Appropriateness of training	✓		
	Group session evaluation	Quality, appropriateness and usefulness of group sessions, engagement/involvement and ongoing support	4 months	8 months	12 months
	Other evaluation	Use of peer leader workbook, participant handbook, implementation fidelity and challenges and barriers	4 months	8 months	12 months

*Abbreviations*: *WHO STEPS* World Health Organization Stepwise approach to surveillance, *PROLIFE* Population registry of lifestyle diseases, *CURES* Chennai urban rural epidemiological study, *MESA* Multi-ethnic study of atherosclerosis, *ENRICHD* Enhancing recovery in coronary heart disease, *HbA1c* Glycosylated haemoglobin, *HDL* High density lipoprotein, *LDL* Low density lipoprotein.

### Step 1: Home screening

The participants receive home visits from the data collectors. After obtaining the written informed consent, a screening questionnaire consisting of eligibility criteria and the IDRS is administered. The IDRS is a diabetes screening tool which creates a score of between 0 and 100, based on age, family history of diabetes, physical activity, and waist circumference [36]. Waist and hip circumferences are measured using a Seca measuring tape (model 201) in a private area of the household in accordance with the WHO STEPS protocol [38]. Briefly, waist circumference is measured over bare skin at the midpoint between the lower margin of the last palpable rib and the top of the iliac crest. Hip circumference is taken at the maximum circumference over the buttocks over light clothing. Participants who meet the eligibility criteria with an IDRS value of ≥ 60 are invited to attend a mobile clinic in their community. Those who are not eligible are excluded from further study participation. Questionnaires are checked for completeness on a daily basis by a local resource person (LRP).

### Step 2: Mobile clinic

The clinics run from 6.00 am to approximately 10.30 am on Saturdays and Sundays. Community buildings such as schools, church halls, *anganwadis* (mother and childcare centre), halls in primary health centres, *panchayat* (local government office in rural areas) halls, and youth clubs are used for organising the clinics. Each clinic is staffed by the project manager or study coordinator, seven data collectors, two laboratory technicians, and one LRP. The participant is registered in the clinic only if they have fasted for 8–12 hours. If not, they are asked to attend the clinic in a nearby PB on another morning. Questionnaires are administered to collect information on socio-demographic, behavioural and psychosocial measures, diabetes knowledge, healthcare utilisation, family history of chronic diseases, and use of medications. Questionnaires are checked for completeness prior to the participant leaving the clinic. Blood pressure (BP) and anthropometric measures (height, weight and bioimpedance) are taken in accordance with the WHO STEPS protocol [38]. Briefly for BP, the left mid-arm circumference is measured using a tape to determine the appropriate cuff size. BP is recorded three times using the Omron automatic blood pressure monitor (model IA2) with a minimum of three minutes between the readings. Height is measured using a Seca stadiometer (model 213) while the participant is standing without headgear and footwear, with feet together, heels against the back board and knees straight, and looking straight ahead [38]. Weight and bioimpedance are measured using a TANITA body composition analyser (model SC330) while the participant is standing still without footwear, with one foot on each side of the scale, facing forward, and arms at their side [38]. BP monitors are calibrated weekly using a sphygmomanometer. Waist and hip circumferences are repeated for 10% of the participants for quality assurance. Blood samples are centrifuged at the clinic and transported to a laboratory accredited by the National Accreditation Board for Laboratories (NABL) [50] for processing. For external quality control, five percent of the blood samples are transported to a laboratory accredited by NABL [50] and College of American Pathologists (CAP) [51]. Participants with blood glucose values in the diabetic range are excluded from further study participation and referred to a government health care facility for further management. Those without T2DM, i.e. those at ‘high risk’ of developing T2DM are invited to continue their participation in the study. Participants complete assessments at baseline, 12 and 24 months.

### Study arms

#### Control arm

Participants in the control arm are provided with a health education booklet containing information on symptoms, complications, and risk factors of T2DM with the recommended levels for primary prevention. Participants are also given a copy of their blood pressure, anthropometric and biochemical measurements collected at baseline, 12 and 24 months with a recommendation to consult a healthcare provider if the values are abnormal.

#### Intervention arm

**Figure 3:**
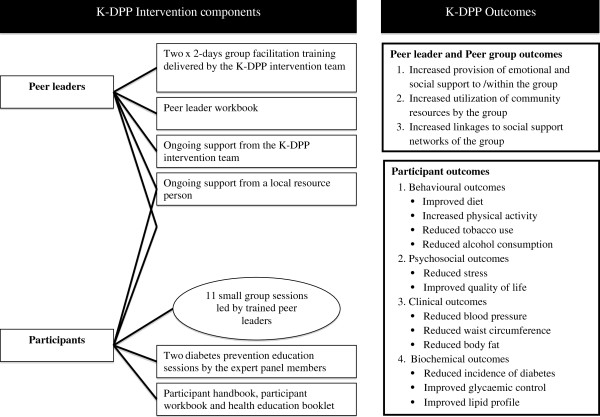
Kerala diabetes prevention program intervention components and outcomes.

This is a multifaceted intervention delivered at multiple levels as summarised in Figure 3. It has been developed and culturally adapted based on our previous work [16-19]. The main mode of delivery is peer-led, small group sessions with training and ongoing support to the peer leaders by the K-DPP intervention team (comprising Intervention manager and Intervention assistant) and practical support from LRPs nominated for each group. Due to low levels of awareness of diabetes and its prevention identified in our needs assessment study, small group sessions are also complemented with two diabetes prevention education sessions (DPES) [52]. Furthermore, an expert panel is formed to meet information needs identified in small groups. The lifestyle intervention has been tested and modified based on a pilot intervention with two groups in 2012–13.

### a. Small group sessions

Small group sessions are planned with specific objectives to support and lead the group in the lifestyle change process. Participants are encouraged and reminded by the LRPs to attend the sessions organised fortnightly till session four and monthly from session five to session 13. Meeting places and times are chosen with the group to maximise convenience. The first session is an inaugural meeting introducing the program and its potential benefits to the participants and their families and is delivered by the K-DPP intervention team. Participants are provided with a participant handbook and participant workbook. The participants are further informed on DPES 1 scheduled within two weeks from the date of the first session. Participants are encouraged to bring family members to the DPES or send a family member preferably their spouse if they cannot attend the session.

Session 2 focuses on setting ground rules for the group and identifies one male and one female peer leader. It also prompts participants to recollect and consolidate the information received from the DPES 1. The next 11 sessions are led by the peer leaders. During the sessions, the group members discuss and share their behaviours, set SMART (Specific, Measurable, Attainable, Relevant, and Time-bound) goals, monitor and review goals. All groups tackle diet and physical activity, but the specific contents of the group sessions are flexible and depend on each group’s needs. Suggested contents for diet cover e.g., portion size, identifying cooking substitutions to reduce fat, increasing fruit and vegetable consumption, and decreasing sugar intake, while contents for physical activity include e.g., finding enjoyable activities for individuals and groups, incorporating those activities into daily routines, and avoiding injuries/accidents. Tobacco control and cessation, reducing alcohol consumption and sleep are dealt with based on the participants’ needs and interest. Furthermore, all groups discuss and identify community-based and other resources that can be utilized to support and sustain these behaviours. Weight monitoring is also performed in the sessions. Peer leaders and the participants are encouraged to participate in social activities such as walking groups and other supports beyond the group sessions.

### b. Diabetes prevention education sessions

DPES 1 and 2 are delivered by expert panel members comprising of specialist advisors on diabetes, nutrition, and physical activity. DPES 1, which is conducted within two weeks after the first small group session, introduces the participants to an understanding of T2DM and its risk factors. The session primarily focuses on development of T2DM and its risk factors, strategies for primary and secondary prevention, misconceptions around T2DM, and the role of lifestyle modification in prevention of T2DM. The participants are also briefed on how the K-DPP and peer support program will help them in breaking the chain of disease for themselves and their family members using community resources.

DPES 2, which is conducted within two weeks after the third small group session, focuses specifically on the risk factors that can be modified including behavior change for diet, physical activity, sleep, alcohol use and tobacco use and its effects on weight, waist circumference, blood pressure and blood glucose.

### c. Resource materials for participants

The participant handbook contains information about peer support, objectives and benefits from attending small group sessions, the role of peer support in assisting with lifestyle modification, principles that guide a group, who are the people at risk for T2DM, and risk factors in detail. The participant workbook guides the participants through 11 peer-led small group sessions with self-monitoring of their lifestyle behaviours, goal setting, goal review and ongoing group support. In addition to the participant handbook and participant workbook, participants also receive the health education booklet that is given to the participants in control arm. Participants are also provided with a non-elastic measuring tape and are taught how to measure their waist circumference to assess the progress towards their goal. The peer leaders of each group are given cups and spoons to discuss with the group how to measure their daily consumption of oil, sugar, salt, rice and vegetables and set targets.

### d. Training and support for intervention delivery

Peer leader training (2 days) is conducted by the K-DPP intervention team and aims to equip the peer leaders with the following skills: group facilitation and communication skills, how to set and monitor goals for lifestyle behaviours, goal setting and planning for healthy lifestyle. Peer leaders are also instructed to maintain records of every interaction with the participants and the K-DPP intervention team. Peer leaders are provided with a peer leader workbook, which describes the objectives of each session with an activity guide and exercises for the peer leader to improve communication and to lead the group effectively in achieving their behavioural targets. In order to reduce the engagement of the K-DPP intervention team with the peer-led sessions, one refresher training (2 days) is given to the peer leaders after session five to share their experiences on conducting sessions and to obtain guidance from other peer leaders. Peer leaders are contacted by telephone before and after each session to reflect on the sessions to be conducted and thereafter to get feedback from the peer leaders on the participation of the group members. Face-to-face meetings with groups of peer leaders are also organised at regular intervals to facilitate ongoing support and communication between the peer leaders and the K-DPP intervention team and among the peer leaders.

Expert panel training (0.5 days) is conducted to inform experts of the study details, the intervention program and the behavioural targets.

Local resource person training (1 day) is conducted to provide details of the program to the LRPs as well as to advise them of the intervention components and discuss their role as a facilitator of the peer-led small group sessions.

Participants are also given a copy of their blood pressure, anthropometric, and biochemical measurements collected at baseline, 12 and 24 months with a recommendation to consult a healthcare provider if the values are found to be abnormal.

### Outcome measures

The primary outcome measure is the incidence of T2DM based on a single 2 hour OGTT. Secondary outcome measures include: behavioural (tobacco use [38], alcohol use [38], physical activity [39], sedentary behaviour [40], diet [41] and sleep), psychosocial (depression [44], stress [45,46], anxiety [47], health-related quality of life [48] and social support [49]), clinical (blood pressure [38], waist circumference [38], body mass index [38] and bioimpedance [38]), and biochemical (glycaemic control [fasting plasma glucose and 2 hour post glucose load, and HbA1c], lipid profile and fibrinogen).

### Program evaluation

Glasgow’s RE-AIM framework [53] is used to report on the program’s reach, efficacy, adoption, implementation and maintenance. For each dimension of the RE-AIM framework, indicators for measurement have been developed, relevant data sources have been identified and the appropriate data collection methods are implemented. Evaluation tools have also been developed to measure different features of the program implementation, including program fidelity, acceptability and feasibility. In the context of wider implementation and scale up in real world settings, Pronk’s PIPE (penetration, implementation, participation, and effectiveness) Impact Metric [54] will also be used to assess the impact of the program from both a program administrative perspective and a user or consumer perspective.

### Economic evaluation

The economic evaluation of K-DPP will assess whether the program offers value for money from the societal perspective, thus includes costs incurred by the government and also participants and their families. Detailed information on healthcare resource use, and out of pocket payments are collected at baseline and each follow-up point. The questionnaires will also collect information on participants’ time away from employment due to ill health. Healthcare resource use will be valued using a primary costing study, given the absence of standardised published prices in India. The analysis will estimate the cost for each participant in the trial, the average cost per participant in each arm of the trial, as well as the total cost of delivering the intervention. The cost of the intervention will include the cost of setting up, delivering and maintaining the intervention. These estimates will be useful for future research assessing the scalability of the program, and the potential budget implications of implementing it into standard practice. Effectiveness will be measured in terms of screening numbers needed to identify one case of ‘high risk’ for developing diabetes and numbers needed to treat to prevent or delay one case of diabetes. Incremental cost-effectiveness ratios will be estimated comparing the costs and outcomes. Sensitivity analysis will be undertaken to test the robustness of the analysis in terms of the cost inputs and health outcomes. Trial participants are also completing the Short Form-36 (SF-36) at baseline and follow-up. These can be converted into utility weights using the SF-6D algorithm [55], which then allow for the calculation of quality adjusted life years (QALYs). Additional economic analyses will be employed to estimate the cost per QALY gained. Currently there is no Indian-specific SF-6D algorithm, but research to generate one is planned. In the absence of a country-specific algorithm, the UK, Australian and Singaporean algorithms will each be used as robustness checks.

### Data analyses

All analyses will be intention to treat, i.e. without regard to the compliance of individuals within their allocated study arm. Multiple imputation methods to infer missing values for FPG and 2 hour post glucose load at follow-up will be used. Analyses will be performed across the imputed datasets and results will be combined using Stata’s “mi” commands. Analyses will adjust for clustering by PB by calculating robust standard errors using the information sandwich formula. FPG at follow-up will be compared between arms by analysis of covariance, adjusting for baseline FPG. Logistic regression will compare 2-year cumulative incidence of T2DM between arms. Cox proportional hazards regression will compare time to diabetes onset between arms. The regression models will include adjustment for baseline measures, when comparing outcomes between arms. All analyses will be performed in Stata statistical software, Release 13.

## Discussion

This paper describes the protocol for a cluster randomised controlled trial of a peer-led lifestyle intervention program to reduce incidence of T2DM among individuals at ‘high risk’ of developing T2DM. The successful implementation of this trial will contribute to improved policy and practice regarding lifestyle intervention programs to prevent diabetes in India and other resource-constrained settings.

### Trial status

The trial is currently in the recruitment phase.

## Abbreviations

T2DM: Type 2 diabetes mellitus; IGT: Impaired glucose tolerance; IFG: Impaired fasting glucose; IDPP: Indian Diabetes Prevention Programme; FPG: Fasting plasma glucose; K-DPP: Kerala Diabetes Prevention Program; CONSORT: Consolidated Standards of Reporting Trials; PB: Polling booth; NCDs: Non-communicable diseases; LACs: Legislative assembly constituencies; IDRS: Indian Diabetes Risk Score; OGTT: Oral glucose tolerance test; WHO: World Health Organization; STEPS: Stepwise approach to surveillance; LRP: Local resource person; BP: Blood pressure; NABL: National Accreditation Board for Laboratories; CAP: College of American Pathologists; DPES: Diabetes prevention education sessions; SMART: Specific, Measurable, Attainable, Relevant, and Time-bound; RE-AIM: Reach, efficacy, adoption, implementation, and maintenance; SF-36: Short Form-36; QALY: Quality adjusted life years; PROLIFE: Population registry of lifestyle diseases; CURES: Chennai urban rural epidemiological study; MESA: Multi-ethnic study of atherosclerosis; ENRICHD: Enhancing recovery in coronary heart disease; HDL: High density lipoprotein; LDL: Low density lipoprotein; PIPE: Penetration, implementation, participation, and effectiveness.

## Competing interests

The authors declare that they have no competing interests.

## Authors’ contributions

TS contributed to the study design, coordinating the recruitment and data collection and prepared the first draft of the manuscript. EDW contributed to the study design, involved in writing the original grant proposal and preparation of earlier drafts. NP is managing the project and participated in preparing the first draft of the manuscript. K-DPP Principal Investigators (BO, KRT, RT, PZ, EF, BH, DJ) and Associate Investigators (PA, RW, PL, AM, JS) were involved in writing the original grant proposal, contributed to the study design, preparation of the manuscript and approved the final draft. EM participated in designing the intervention and preparation of the manuscript. ZA contributed to the program evaluation design and preparation of the manuscript. MD contributed to the study design, participated in writing the original grant proposal and preparation of the manuscript. All authors read and approved the final manuscript.

## Pre-publication history

The pre-publication history for this paper can be accessed here:

http://www.biomedcentral.com/1471-2458/13/1035/prepub
